# Notes and Comments

**Published:** 1924-05

**Authors:** 


### NOTES AND COMMENTS.

### THE BRITISH EMPIRE EXHIBITION.

THE great British Empire Exhibition at Wembley, which was opened, amid stately magnificence, by the King on St. George's Day, is the most remarkable "exposition" that has ever been held. Our fathers and grandfathers dated many things from the Great Exhibition of 1851. The world is now too full of remarkable events and we live too fast for us any longer to date anything save from the war; but this wonderful Imperial Fair cannot but stir the pulse and inspire the patriotism of the whole Empire. It emphasizes the boundless resources of "all the Britains" and marks its industrial and commercial progress as nothing else could do. But it is not all materialism, and, as the King pointed out in his speech at the opening ceremony, important sections of it are devoted to showing what we have done, are doing, and hope yet to do in all matters connected with the public health. There are to be seen illustrations and models of town-planning and housing, water supply, sewage disposal, public cleansing and other departments of municipal sanitary work, to say nothing of diagrams, charts, and photographs recording our efforts for the prevention of disease and the saving of infant life. A careful study of this department of the Exhibition will probably have many surprises for that large section of the people which takes all these things for granted and recks little of the labour and devotion which have brought them about. A quickened interest in many matters affecting the health of the nation may not unreasonably be expected from this closer acquaintance with the work of the army of men and women and the multitude of public departments which for half a century past have been labouring to make a healthy people and a salubrious land.

### MORE HOUSING SCHEMES.

THE month of April has been a period of febrile activity in everything connected with the urgent subject of the housing of the people. Four different Bills relating to rent restriction and evictions for non-payment of rent have been brought into the House of Commons, and, oddly enough, that prepared by the Government was the most impossible of the series. It was based on the familiar theory that the house-owner is an enemy of the human race, and left him to bear the loss in cases where the tenant was unable to pay in consequence of unemployment. The finger of the Glasgow extremists was plainly to be seen in the measure, of which the House made short work, and in the end the Cabinet has adopted and amended a private member's Bill under which the rates are to provide the landlord with his rent. There is abundant room for doubt whether unemployment accounts for most of the unpaid rent, since the number of men in work is steadily rising. Meanwhile the Government appear to be on the point of adopting a scheme drawn up by the employers and employed of the building trades, for providing two and a-half million houses in the next fifteen years. In return for a guarantee of employment during that period the Unions will allow a certain proportion of dilution by the extension of apprenticeship. The concession is being bought at too high a price. The apprentices will be too few, and experts calculate that the plan is based on a very low average of output. But a serious objection is that we shall not need two and a-half million houses in the next decade and a-half. The population does not increase at so high a ratio and, even allowing generously for arrears and rebuildings, we could not people the houses if we had them. Every house municipally built will show a loss of six shillings a week, of which the rates will pay two and the taxes four, and when the scheme is completed the rates will be increased by an average of tenpence in the pound. But it never can be completed—it is all too grandiose. It would have been far better to leave Mr. Neville Chamberlain's Act of last year to continue the work it was steadily doing. It is much to be feared that the encouragement it gave to private enterprise will now be replaced by a fatal lack of confidence.

### STANDARDISING SUMMER TIME.

NOTWITHSTANDING the Second Reading which the House of Commons has given to Sir Kingsley Wood's Summer Time Bill, the congestion of Parliamentary business is so serious that it by no means follows that it will reach the Statute Book. The measure seeks to place Summer Time on a permanent basis and to make it run from the first Sunday in April to the first Sunday in October. This would not only carry out the decision reached by the recent conference of representatives of England, France and Belgium, and so bring us into line with the dates adopted by our two nearest Continental neighbours, but would make an end of the absurdity of constantly re-enacting Summer Time. The subject has a definite and important bearing upon the public health. The addition of an hour to daylight during one half of the year has had excellent results, and the opponents of the change have never succeeded in controverting the declaration of the British Medical Association that "Summer Time is beneficial to the health of the nation." That the extra allowance of daylight is injurious to children has been shown by experience to be a myth. It is true that cows have made it known (by their next friend) that they do not approve of it, for cows are conservative "die-hards" who resist all change on principle. But, with the occasional exception of those with crumpled horns, they are notoriously mild and agreeable, and even under the provocation of Summer Time are unlikely to take to evil courses, such as tossing the milkmaid or kicking over the milk-pail. Like payers of income-tax they sacrifice themselves for the public weal.

### THE PART-TIME MEDICAL OFFICER.

WHEN should a community cease to be satisfied with a part-time medical officer? The question has recently been pressing itself upon Enfield, and, as population grows, it will press itself upon many other places. In a really small community there is, generally speaking, no need for a head sanitary official's whole time, though we have no doubt that, owing to local circumstances, even some small places would be the better for the close supervision of a health specialist. Directly, however, a small town begins to " develop," as the horrid expression goes, its sanitation becomes a matter of supreme importance, and the few extra hundreds a year required for the salary of a full-time M.O.H. should be a first charge upon the growing rateable value. A clean bill of health is a splendid asset for any town, and it is often found that the bigger the town the better its health. The inference is obvious, and there is little room for doubt that part-time medical officers will be much less numerous in the future. These officers have done, and continue to do, admirable work which has had a demonstrable influence in improving the health of those whom they serve, and it would be absurd to displace them for the mere sake of change. But the M.O.H. is rapidly becoming a specialised man of science, and the greater the agglomeration of people in towns the more necessary will it be to multiply the number of men who, upon a solid professional foundation, graft the distinctive training needed for work to which no knowledge comes amiss.

### INFLUENZA AND INFANT MORTALITY.

COINCIDENTALLY with the valuable article upon infant mortality which Sir Arthur Newsholme contributes to our issue this month come some disquieting figures showing the influence upon the infant death-rate of the epidemic of influenza which recently passed through Western Europe. At the end of January, 1923, London's infant mortality rate was 62 per thousand living births, a ratio beaten only by twenty-six Swiss towns with an average of 48, and San Francisco with 61. Exactly a year later London was losing 81 small children per thousand, and at the end of February, when the epidemic was at its height, the loss mounted to 114. The infant death-rate of the first quarter reached 97 per thousand in London and 107 in the great towns. Nor did the birth-rate provide any compensation—quite the contrary, indeed, for the quarter shows the record low-water mark for births in London, the war years only excepted. When Nature puts some limit upon the increase of an over-populated country such as this, we need not repine; but that disease should sweep away so large a proportion of the little lives that actually come into being is grievous in the extreme. At present influenza is one of our conquerors—one, too, that has long been accustomed to conquest; but as the villain of melodrama used to exclaim, " The day will come ! "

### THE DOGS BILL.

THERE is abundant reason for congratulation that the Dogs Protection Bill introduced by Lord Banbury of Southam has been decisively rejected on Second Reading by the House of Lords. In an admirable and incisive speech Lord Knutsford riddled the arguments for the measure and laid stress upon the intolerable restrictions which would have been placed upon research and experiment had it been passed. Dogs are fully protected from the infliction of pain and suffering by the existing law, and Lord Banbury's Bill would have made it impossible to use them for experiments into which vivisection does not enter, but which are of essential moment for the reduction of disease and the saving of human life. The relations between the nutritive values of butter and margarine were ascertained by experiments which Lord Banbury would have forbidden; we have learned how to conquer rickets by experiments upon puppies; insulin was discovered by Dr. Banting, as Lord Knutsford said, " solely and only by means of operations and experiments on dogs," yet Lord Banbury would condemn him as a monster of inhumanity. " Instead of which " he has been given the Nobel Prize as one of the world's benefactors. Researches and experiments are now being made by the aid of dogs which are exceedingly likely to be of as much benefit to them as other experiments have been to human kind. Distemper is a characteristic and often fatal disease of dogs, and many a valuable, faithful, and affectionate animal will, we may hope, be saved by means of the Distemper Fund which we owe to the energy and initiative of Sir Theodore Cook, the Editor of the *Field*. For reasonable people it should be enough that the Medical Research Council has roundly declared that the prohibition of experiments upon " the friend of man " would place " an insuperable and permanent barrier " across some of the chief paths of its work. We have every respect for Lord Banbury's motives, but we rejoice in his defeat, and we hope that no more will be heard of measures such as that which the Lords have so happily consigned to limbo.

### PIGMENTS IN ANIMAL LIFE.

IT is common knowledge that the most important pigment in vertebrate life is hæmoglobin, the red pigment of the blood which carries oxygen from the lungs to the tissues, and it is easily realised that there is a close analogy in composition and function between this red hæmoglobin and the green chlorophyll of plants. But Professor Barcroft, in his recent lectures before the Royal Institution, carried us a stage farther. Nature seems to have bred, as it were, from a single pigment-producing substance a whole flock of chemical entities, each vital to the widely different needs of the particular living thing which contains it. Substances such as hæmoglobin appear to consist of two parts, the one containing the vien in this case and being the essential producer of the colour and oxygen-carrying power, and the other a dead weight of albuminous material to which it first is attached. By varying the proportions of the two, nature has succeeded in providing a wide range of circulatory fluids, blood and its analogues, which show a great range of power to carry and retain gases. By such variation it has been possible to meet the respective needs in gas absorption of such widely different creatures as, say, the daisy, the fish, the earthworm, and the gazelle. Not all possess hæmoglobin, of course. Chlorophyll in plants has already been mentioned. But there are other allied pigments. The cuttle-fish has blood which is sometimes blue. Human blood enters the lung purple and leaves it red; that of the cuttle-fish enters the gill colourless and emerges blue. In certain star-fishes the blood corpuscles are said to be of various colours, brown, green, lemon-yellow and indigo-blue. The brown cells turn green when deprived of oxygen and would appear in these animals to be the cells concerned in respiration. In many of these molluscan pigments the metal is copper instead of iron, as in human blood, a fact doubly striking when we realise that copper is to man a moderately active poison.

### "THE STAFF OF LIFE."

WE observe with satisfaction that Mr. A. R. Tankard, the City Analyst of Hull, is continuing his efforts to bring home to the public the perils of adulterated or chemically treated flour. He points out that poor flour which, by the aid of chemistry, is made to look better than it is and to acquire better baking possibilities, is causing eczema on the hands and arms of the bakers who use it. It is said, we know not upon what authority, that 90,000 sacks of this deleterious rubbish are made into bread in London alone, but we need not pin ourselves to quantities to be aware that much harm is caused to the digestion by the eating of bread made from what is really impure flour. Food which will produce eczema on hard skin is obviously dangerous to delicate membranes. So far as we know, the only object of thus "improving" poor flour is to put money into the pockets of the millers. Sir H. C. Munro's Ministry of Health Committee which is inquiring into the use of preservatives and colouring matter in food, is necessarily concerning itself with this important side of the subject, and we trust that its report, when it comes, will be speedily followed by a stern strengthening of the law against adulteration. England is especially unfortunate about its bread. The sodden, half-baked stuff which most of us are compelled to eat, for want of anything better, has much to answer for in the matter of the national indigestion. We are a dyspeptic people, and we shall continue to be so as long as we eat bread from which the nutritious and digestive ingredients have been carefully removed, either to put money into somebody's pocket, or to gratify the absurd fancy for dead-white bread. Until a wholesome loaf is forthcoming we shall be wise to eat brown bread, though even that is not always well baked or as well constituted as it ought to be.

### STARVING THE NURSE.

IT would be difficult to find any class of people which does more valuable work for the public health than health visitors and school nurses. Yet, as Miss Rundle, the Secretary of the College of Nursing has just been reminding the country, we are doing our best to starve those who are already in function and to dry up future sources of supply. School nurses and health visitors are public officials, appointed and paid by local governing bodies, yet the average salary offered to them is £150 a year, rising to a maximum of £180. When imperative elementary expenses such as board and lodging and clothes have been provided for the scanty remnant of such a guerdon is clearly insufficient to pay for any form of insurance, to buy books necessary for keeping abreast of professional knowledge, or to allow of a holiday. Yet the devoted women whom we treat so scurvily have to be skilled and certificated, to possess abundant tact and sympathy, and to bear a heavy physical as well as a constant mental strain. Without them the medical officers of health would assuredly have failed to halve the infant death-rate. That marvellous work has been accomplished in the main by the instruction the public health worker has been taught to give to parents and by her readiness to place her trained intelligence and technical knowledge at the disposal of those who, before, had the haziest notions of the meaning of healthy living or of how to feed, nurture and rear children. A robust body, an alert mind and a high standard of education are needed for such duties as these, and, to speak plainly, it is a scandal that women of this type should be subject to constant anxiety about ways and means and deprived of all opportunity of making even the scantiest provision for invalidity or old age.

### VENEREAL DISEASE CAMPAIGN IN GERMANY.

A PAPER in a recent number of the leading German medical journal, *Deutsche Medizinische Wochenschrift* makes sad reading. During, and immediately after, the Great War, a very complete organisation for the control of the venereal diseases was established, with the dispensary as the centre. These dispensaries provided not only diagnosis and advice, but also supervision and treatment. They took a leading part in the administrative control of the venereal diseases, helping to secure notification, to conduct educational propaganda and in various other ways to restrict the opportunities for dissemination of these diseases. By 1919 there were as many as 138 such dispensaries in full activity, and it is calculated that their *clientèle* were numbered by the hundred thousand. In 1922 alone one such dispensary carried out 3,385 first examinations and 5,271 subsequent ones, providing treatment in 4,602 cases. The cost of these dispensaries was distributed over the central and local authorities and insurance societies. With the collapse of the mark and the immediate prospect of bankruptcy, the central authorities have of late disowned one obligation after another in connexion with the maintenance of these dispensaries. Such a policy is, of course, infectious, and it would seem that there is a serious prospect of this efficient and complicated machinery being more or less completely scrapped for want of money. Since the venereal diseases constitute an international problem, this state of affairs in Germany is one which no other country in Europe can afford to regard with equanimity.

### DRUNK FOR THREEPENCE.

TO be able to get drunk for threepence, and "gloriously" drunk for three days for sixpence, is an ideal incomprehensible to persons who have not had their brains poisoned and their wills weakened by the constant swallowing of alcohol. It would appear that methylated spirit is almost, if not quite, alone in producing the extremity of drunkenness at the cheapest possible rate, and it has been decided, by no means too soon, to put a drastic end to the growing addiction to a drink which in America and on the Continent bears the much more alarming name of "alcohol." From May-day it will be treated with a substance designed to make it too horrible to drink. Its colour is already far from inviting, and it is earnestly to be hoped that it really will now be made impossibly nauseous. But we must not be too sure. When what is sought is mere oblivion irrespective of the means employed, depraved human nature is capable of swallowing exceedingly nasty things. Yet it would be unfortunate if a troublesome control should be necessary over the sale of a liquid which is of real value for many legitimate household purposes. Methylated spirit has a peculiar fascination for the female drunkard, who usually prefers getting fuddled in secret, and the number of convictions of women for drunkenness by its means has steadily grown, especially in Scotland. There is no need to emphasize the familiar fact that the ultimate consequences of addiction to this vice are in many respects much worse than those which wait upon the excessive consumption of alcohol in its more convivial forms.

### MATERNAL MORTALITY.

IN England and Wales there are not less than 700,000 mothers giving birth to children every year, and of this number approximately 3,000 per annum have died during the last ten years in the fulfilment of their maternal functions. Far more than 3,000 of the mothers who survive are permanently injured or invalided. As Sir George Newman, in his Prefatory Note to Dr. Janet Campbell's Third Report on "Maternal Mortality" points out, "this is a serious and largely avoidable loss of life at the time of its highest capacity and in its most fruitful effort." In this respect we compare unfavourably with several European countries, and the remarkable decline in infant mortality has not been accompanied by any appreciable decline in maternal mortality. The main cause of these deaths is puerperal infection, and its excessive rates are, generally speaking, found in rural districts and in areas engaged in textile manufacture and coal-mining. Dr. Campbell states that "insanitary conditions appear to have a less injurious effect upon maternity than might be supposed," but adds that "domestic uncleanliness and lack of facilities and conveniences necessarily add enormously to the difficulty of applying effectual antisepsis and to the risk of operative procedure." Much remains to be done in the way of ante-natal supervision—indeed, it is clear that no solid progress can be made apart from it, and there is abundant room for more co-operation between doctor and midwife, and between both and the Local Authority. The Report makes distinctly disquieting reading, since it shows that the steady improvement in the nation's health has not extended to some of its most valuable members.

### A "CLEANLINESS WEEK."

THE "Week" idea has so captivated the imagination and given birth to so great a variety of "Weeks" that the time would seem to be not far distant when there will be as many "Weeks" in the year as there are saints in the calendar. The latest "Week" idea comes from Christiania, where the "Sanitetsforening" Public Health Association proposes to celebrate the 300-year anniversary of the town by organising a "Cleanliness Week" some time in May. Perhaps a "Rubbish Week" would be a more appropriate title for this scheme, the chief object of which is to organise a *razzia* throughout every cellar and attic of the town's 8,000 houses, with a view to getting rid of all lumber and rubbish. The "Sanitetsforening" has solicited the co-operation of various other organisations including the Board of Health. This body has approved of the scheme in principle, but has ventured the forecast that it would cost about £4,000 to dispose in a week of all the rubbish. This estimate is evidently based on the assumption that the flotsam discharged by the Christiania houses during rubbish "Week" would be of no value, but with a little discrimination this should certainly not be the case, and it is even conceivable that such a Week might be made as profitable as the conventional Jumble Sale in this country. What is rubbish in one house may be prized goods and chattels in another, and there are few things which cannot be turned to some use. Dirt has been defined as "matter out of place," and with rubbish, as with dirt, it is only a matter of finding the right place for it.

### THE PREVENTION OF CONGENITAL SYPHILIS.

THOUGH the rather fussy interest taken in the venereal diseases during and after the war seems to be collapsing, there is no reason to suppose that the decline of these diseases is equally rapid. In the case of syphilis there has, indeed, been a very notable decline of late in such countries as Belgium and Denmark, where the campaign against it has been well organised. But the problem of syphilis, notably congenital syphilis, is still far from being satisfactorily solved. A noteworthy step in this direction has lately been taken by Professor Almkvist, of Sweden, whose recommendations are supported by convincing statistics. In his opinion a doctor should examine every pregnant woman for syphilis, however negative her history or career may seem to be, when she comes to him for the first time. He should examine her, not only in the ordinary way, but should also submit a specimen of her blood to a serological examination. Should the blood give a positive Wassermann reaction, salvarsan and mercury should be given alternately till the birth of the infant, and for some time afterwards if necessary. Among Professor Almkvist's syphilitic patients there were twenty-eight who did not enjoy anti-syphilitic treatment before they were confined. Only in one of these cases did the infant fail to show signs of syphilis; but among the thirty-nine syphilitic mothers who were given anti-syphilitic treatment before confinement there were only four whose infants showed unmistakable signs of disease, and it was probable that in these four cases the treatment had been inadequate. These figures clearly show that congenital syphilis is a disease easily preventable, provided the mother's affection is diagnosed early. But that is the rub. Many expectant mothers do not know that they are suffering from syphilis, and many of them would be indignant were they told that their blood was to be examined for evidence of this disease.

